# A Digital Twin Lake Framework for Monitoring and Management of Harmful Algal Blooms

**DOI:** 10.3390/toxins15110665

**Published:** 2023-11-17

**Authors:** Yinguo Qiu, Hao Liu, Jiaxin Liu, Dexin Li, Chengzhao Liu, Weixin Liu, Jindi Wang, Yaqin Jiao

**Affiliations:** 1 Key Laboratory of Watershed Geographic Sciences, Nanjing Institute of Geography and Limnology, Chinese Academy of Sciences, Nanjing 210008, China; liujiaxin12C@163.com (J.L.); adtubeno1@163.com (J.W.); ttoa0718@163.com (Y.J.); 2 Powerchina Zhongnan Engineering Corporation Limited, Changsha 410014, China; 02245@msdi.cn (H.L.); 03453@msdi.cn (D.L.); 03105@msdi.cn (C.L.); 03424@msdi.cn (W.L.); 3 Hunan Provincial Key Laboratory of Hydropower Development Key Technology, Changsha 410014, China; 4 School of Marine Technology and Geomatics, Jiangsu Ocean University, Lianyungang 222005, China; 5 School of Surveying, Mapping and Geographical Sciences, Liaoning Technical University, Fuxin 123000, China

**Keywords:** digital twin lake, water environment management, harmful algal blooms, video monitoring, satellite remote sensing

## Abstract

Harmful algal blooms (HABs) caused by lake eutrophication and climate change have become one of the most serious problems for the global water environment. Timely and comprehensive data on HABs are essential for their scientific management, a need unmet by traditional methods. This study constructed a novel digital twin lake framework (DTLF) aiming to integrate, represent and analyze multi-source monitoring data on HABs and water quality, so as to support the prevention and control of HABs. In this framework, different from traditional research, browser-based front ends were used to execute the video-based HAB monitoring process, and real-time monitoring in the real sense was realized. On this basis, multi-source monitored results of HABs and water quality were integrated and displayed in the constructed DTLF, and information on HABs and water quality can be grasped comprehensively, visualized realistically and analyzed precisely. Experimental results demonstrate the satisfying frequency of video-based HAB monitoring (once per second) and the valuable results of multi-source data integration and analysis for HAB management. This study demonstrated the high value of the constructed DTLF in accurate monitoring and scientific management of HABs in lakes.

## 1. Introduction

Harmful algal blooms (HABs) caused by lake eutrophication and climate change have become one of the outstanding water environment problems in the world [1,2]. HABs can alter the bacterial community structure and disrupt recreation and human health [3]. Unique microbial communities are assembled during a cyanobacterial bloom via stochastic and potentially deterministic processes, including competition, mutualism and trade-offs [4,5]. In recent years, HABs have occurred in over 40% of the lakes with areas over 500 km^2^ in the world, causing serious eco-environmental harms [6]. From May to June in 2007, a large-scale HAB broke out in Lake Taihu and led to a water crisis in Wuxi City [7]. In September 2013, a serious HAB event occurred in the west of Lake Erie, which resulted in nearly 2000 people in Carroll Town, Ohio being unable to access clean drinking water [8,9,10]. Moreover, a multi-day recommendation of “do not drink” was issued in Toledo City for over 500,000 people [11,12]. Considering this background, timely and comprehensive data on HABs in lakes are of great significance for their scientific prevention and control, because emergency response measures can be initiated timely and the harm caused by HABs can be minimized to the greatest extent.

Monitoring methods for HABs in lakes have been widely researched in the past two decades, aiming to obtain both the area and intensity of HABs [13]. Field monitoring is a traditional means for monitoring HABs in lakes. In this kind of method, water samples were collected in the field and then analyzed in laboratory to obtain detailed characterization factors of HABs, e.g., Chl-a concentration and algal density. The present conditions of HABs in the whole lake can be finally assessed with spatial interpolation methods [14,15]. Although these methods have many advantages such as detailed monitoring parameters, high monitoring accuracy, etc., they consume a large amount of manpower, material and financial resources [16]. In addition, the monitoring efficiencies of these methods are quite low and thus they are suitable only for small lakes and reservoirs [17]. To realize efficient monitoring of HABs, many in situ monitoring systems were constructed in lakes [18,19]. With these systems, HAB information and main water quality parameters in core positions were collected continuously and the working costs were reduced. These methods, however, cannot accurately reflect present conditions of HABs in the whole lake with a limited number of monitoring stations [20,21]. More importantly, the 3D spatial distribution of HABs and water quality in the lake body was hard to assess due to the lack of an effective framework for data integration and representation. In recent years, satellite remote sensing has been considered as a new and efficient means for HAB monitoring due to its strong ability to enable large-scale and long-term observations of surface elements [22,23]. In this type of method, both area and spatial distribution of HABs in lakes can be obtained rapidly and periodically via processing methods for satellite imageries such as visual interpretation [24], watercolor inversion [25,26], color space analysis and supervised classification [27,28], spectral index analysis [29,30], etc. The generated HAB products greatly improved the efficiency of HAB monitoring in lakes, but there were still several significant limitations. Firstly, the method was limited by the revisit cycles of the satellites and the uncontrollable meteorological conditions, and high-frequency HAB monitoring was hard to realize during emergency prevention and control periods of HABs [31]. Moreover, the method is limited by the spatial resolution of the satellite imageries, and HAB information regarding the local, sensitive water areas in lakes, such as water sources, cannot be obtained effectively [17]. In order to make up for the deficiencies of the field monitoring methods, in situ monitoring and satellite remote sensing, video monitoring has been gradually applied in HAB monitoring in lakes because it can work continuously without artificial participation. With this kind of means, coverage ratios of HABs in key nearshore areas can be obtained using methods for digital image processing and machine learning [32,33]. However, there were still shortcomings in terms of efficiency. Although the monitoring frequency can be improved to a certain extent with multi-threading technology [17], real-time monitoring was still difficult to realize due to complex network environments in field. More importantly, this kind of means can only monitor HABs in nearshore areas of lakes, while the intensity and spatial distribution of HABs in the whole lake cannot be understood. For HABs monitoring and management, the integration, visualization and analysis of multi-source monitoring data are quite valuable for present condition assessment and abnormity recognition. Traditional research, nevertheless, generally cannot meet this need.

Digital twin, a new leading-edge technology which can accurately reflect physical objects in the digital world by integrating various types of 3D model data such as 3D terrains, 3D real-scenes and 3D entities [34,35], has developed rapidly in recent years, providing new thoughts for the integration, representation and analysis of multi-source HAB data in lakes. In theory, real information of HABs in lakes can be represented with digital twin technology. The existing research about digital twin has focused mainly on medical analysis [36], industrial design [37], urban management [38], etc., and digital twin scenes have been constructed by integrating multi-source 3D model data, multi-sensor monitoring data and multi-variate mechanism models. The objectives of realistic expression and intelligent manageability have been achieved preliminary. However, few works about digital twin technology have been completed at present that focus on lake water eco-environment management. Accordingly, the intelligent level of HAB prevention and control in lakes is still very low.

In summary, the main shortcomings of traditional methods of HAB monitoring, prevention and control contain three main aspects. Firstly, real-time monitoring of HABs in nearshore areas of lakes was difficult to realize and there was still much room for efficiency improvement. Moreover, the water environment of lakes was hard to be realistically represented and precisely analyzed with a limited number of monitoring equipment. Additionally, it was quite difficult to comprehensively grasp the information on the HABs and key water quality parameters in lakes due to the lack of effective methods for integrating multi-source monitoring data. To make a breakthrough in this field, we constructed a digital twin lake framework (DTLF) in this paper for the prevention and control of HABs. The novelty of this work can be summarized as follows:

(1) Browser-based front ends, instead of programs deployed on network servers as usual, are used to execute the video-based process of HAB monitoring. As a result, the problem of low efficiency in the traditional methods is completely solved;
(2) DTLF is constructed by modelling a precise 3D model of the lake body, and the water environment of the lake can be realistically represented using monitoring data on the water quality, as well as precisely analyzed in a 3D manner;
(3) Based on the constructed DTLF, present conditions of HABs and water quality in lakes can be grasped comprehensively by integrating monitoring data from satellite remote sensing, video devices and in situ stations.

## 2. Results

### 2.1. Research Area and Data Source

**Figure 1:**
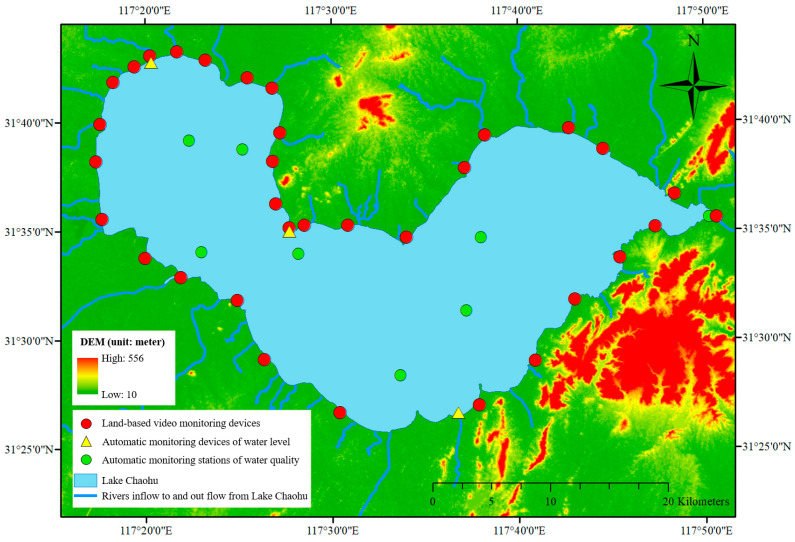
The general situation of the research area (i.e., Lake Chaohu).

The constructed DTLF has been applied in the fifth largest freshwater lake in China, i.e., Lake Chaohu. Lake Chaohu (31°25′28″~31°43′28″ N, 117°16′54″~117°51′46″ E), one of the three key lakes for eutrophication control in China, is located in the center part of Anhui Province. In the past two decades, HABs have occurred frequently in Lake Chaohu [17,31,33], which has become a major issue restricting the sustainable development of the regional society and economy. To prevent and control HABs, many monitoring devices, e.g., automatic monitoring stations of water quality, land-based video devices, etc., have been deployed in and around Lake Chaohu in recent years. Data sources for DTLF construction and HAB monitoring and management are descripted as follows:

(1) Digital elevation model (DEM) of Lake Chaohu Watershed with a spatial resolution of 5 m (Figure 1) was used to dynamically determine the boundary and surface of Lake Chaohu, as introduced in step (1) in Section 5.1;

(2) Forty-two land-based video devices around Lake Chaohu (Figure 1) were used for real-time monitoring of HABs in nearshore areas. From 8 a.m. to 6 p.m. every day, coverage ratios of HABs in key nearshore areas were calculated automatically and periodically (once per second), as introduced in Section 5.3;

(3) Eight automatic monitoring stations for water quality in Lake Chaohu (Figure 1) were used to obtain key water quality parameters for different water depth such as total phosphorus (TP), NH_3_-N, chlorophyll-a (Chl-a), etc., in core positions. The time interval for this monitoring is four hours. Based on these dynamical and layered monitoring data, spatial distribution and multi-dimensional analysis of water quality were realized in the whole lake body, as explained in Section 5.4;

(4) Three automatic monitoring stations for water level (Figure 1) were used to obtain the hourly dynamic water levels of Lake Chaohu. By averaging the monitoring data from the three stations, the final water level was obtained and used to dynamically determine the boundary and surface of Lake Chaohu, as introduced in step (1) in Section 5.1;

(5) 3D underwater topography of Lake Chaohu with a scale of 1:2000 was used to generate the 3D model of the underwater topography, as introduced in step (2) in Section 5.1.

### 2.2. Satellite Remote Sensing of HABs in the Whole Lake

**Figure 2:**
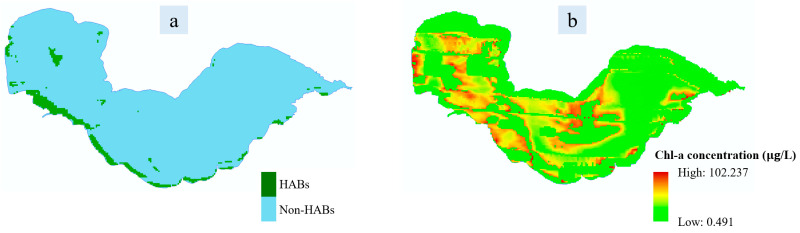
The results of HAB monitoring with satellite remote sensing. (**a**) is the result of HAB monitoring (the accurate area of HABs is 6.51 km^2^), and (**b**) is the inversed result of the spatial distribution of Chl-a concentration. Note that the monitoring date is 17 September 2021 and the satellite data source is Aqua/MODIS.

HAB information, including both area and spatial distribution, was obtained automatically based on the satellite-based monitoring methods introduced in Section 5.2. The used satellite data sources include Terra/Aqua MODIS, Sentinel-2 MSI and GOCI. An example of the satellite-based HAB monitoring is shown in Figure 2, and the accuracy evaluation results is given in Section 2.5.3.

### 2.3. Video-Based Real-Time Monitoring of HABs in Nearshore Areas

**Figure 3:**
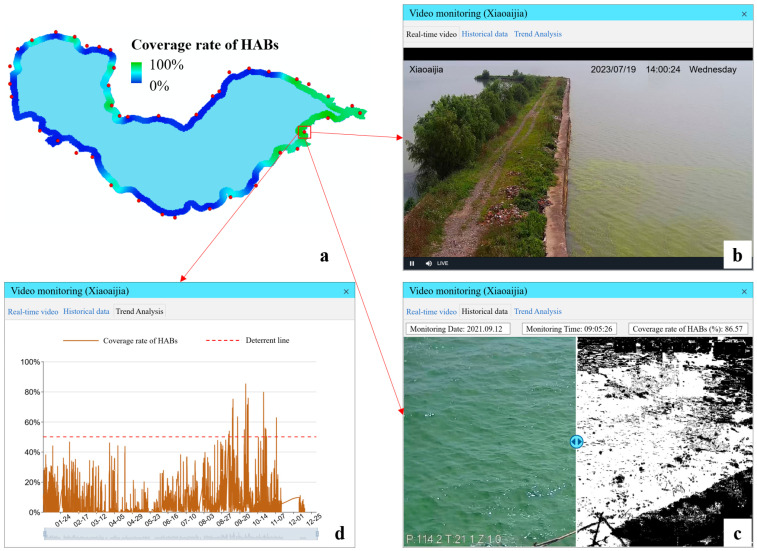
The results of HAB monitoring with video devices around Lake Chaohu. (**a**) is the present situation of HABs in the whole nearshore area, (**b**) is the real-time live situation in field, (**c**) is the monitoring result of HABs, and (**d**) is the long-term changing information of HABs.

There are forty-two land-based video monitoring devices around Lake Chaohu (Figure 1 and Figure 3a). From 8 a.m. to 6 p.m. every day, coverage ratios of HABs were monitored with each video device automatically and periodically (once per second). And the processes were executed only using browser-based front ends. With these land-based video devices, the real-time live situation (Figure 3b), the latest coverage ratio of HABs (Figure 3c) and the long-term changing information of HABs (Figure 3d) were obtained. After obtaining the coverage ratios of HABs via the forty-two video devices, the present situation of HABs in the whole nearshore area of Lake Chaohu was generated with the Kriging Interpolation method (Figure 3a) and updated every second.

### 2.4. Integrated Representation and Analysis of Multi-Source Monitoring Data

**Figure 4:**
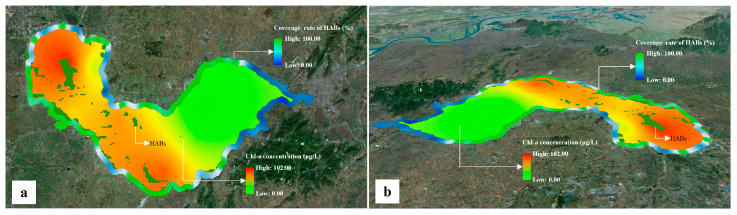
The results of multi-source HAB information integration and expression. (**a**,**b**) are the visualized results from the vertical view and the perspective view, respectively.

**Figure 5:**
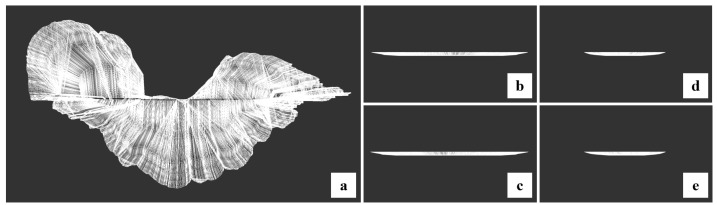
The results of DTLF construction. (**a**–**e**) are the visualized results of the DTLF from the vertical view, the front view, the back view, the left view and the right view, respectively.

**Figure 6:**
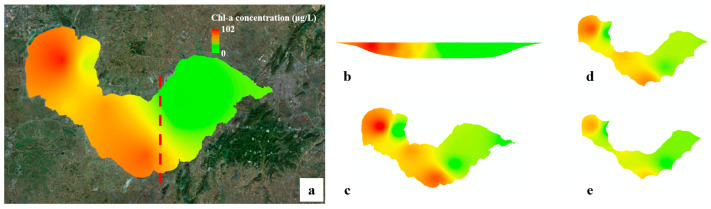
The results of the multi-dimensional analysis of water quality in the constructed DTLF, and the Chl-a concentration is taken as the example parameter. (**a**) is the visualized result of the DTLF from the vertical view, and the red dashed line denotes the section position (the section analysis results is shown in (**b**)). (**c**–**e**) are the layered analysis results of various water depths, i.e., 0.5 m, 1.0 m and 1.5 m, respectively.

Based on the constructed DTLF, HAB information acquired with satellite remote sensing, land-based video monitoring and in situ monitoring was integrated and expressed in a 3D manner (Figure 4). HAB information in the whole lake, key areas and core positions can be grasped and visualized simultaneously. Moreover, based on the constructed DTLF (Figure 5), layer representation and section representation of water quality in the lake were achieved effectively (Figure 6). This can help managers of the lake water environment to understand the 3D spatial distribution of water quality in the lake, from both horizontal and vertical views.

### 2.5. Evaluation Results of Efficiency, Effect and Accuracy

#### 2.5.1. Efficiency Evaluation

The efficiencies of HAB monitoring with satellite remote sensing and video monitoring were evaluated in this paper. The used server configuration is shown in Table 1, and the results of the efficiency evaluation are shown in Table 2, indicating that the efficiencies of HAB monitoring in this paper are quite satisfying, and can fully meet the requirements for HAB management.

#### 2.5.2. Effect Evaluation

It can be inferred from Figure 4 and Figure 6 that the designed functions, i.e., multi-source data integration, visualization and analysis were all implemented based on the constructed DTLF. Actually, the DTLF provided new techniques for lake water quality analysis such as layer representation and section representation that were difficult to achieve in traditional research. In summary, the results of the effect evaluation are quite satisfying.

#### 2.5.3. Accuracy Evaluation

**Figure 7:**
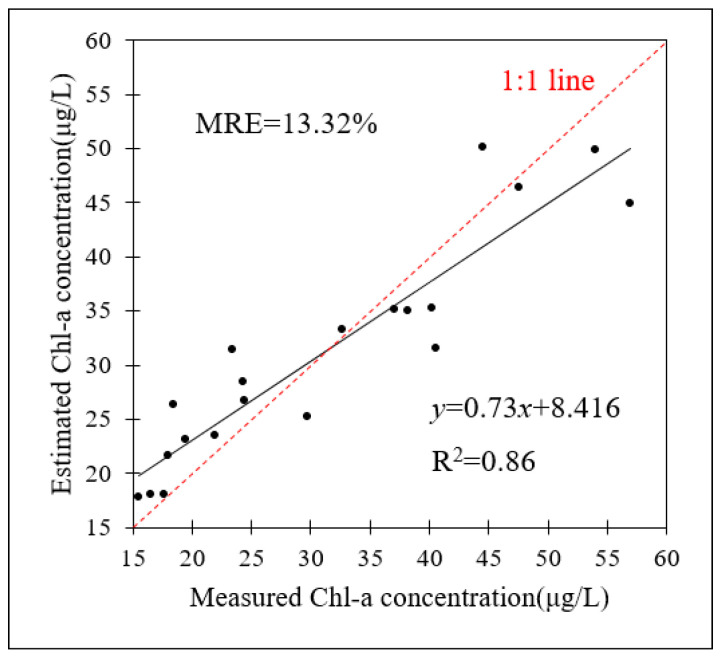
Accuracy evaluation results for Chl-a concentration monitoring with satellite remote sensing.

Twenty points in Lake Chaohu were chosen to evaluate the accuracy of Chl-a concentration inversion using satellite remote sensing. The accuracy evaluation results are shown in Figure 7, indicating that the accuracy is high (MRE = 13.32% and R^2^ = 0.86).

Two thousand images captured with land-based video devices, i.e., validation data that do not participate in the training of the HAB monitoring algorithm, were used to evaluate the accuracy of HAB monitoring based on the video devices. The evaluation method will be introduced in Section 5.5. The evaluation result (F = 0.8552) indicated that the land-based video monitoring method can be used for HAB monitoring in lakes.

## 3. Discussion

### 3.1. Advantages of DTLF

The DTLF design and application in Lake Chaohu demonstrated its high value in the monitoring and management of HABs in lakes. Firstly, different from traditional monitoring methods for HABs [14,18,24], this work aims to provide an efficient way to obtain the real-time situation of HABs in nearshore areas of lakes. From 8 a.m. to 6 p.m. every day, coverage ratios of HABs in key nearshore areas can be accurately and frequently monitored (Figure 3). Supported by this function, the main drawbacks of the traditional inspection work for HABs in lakes, which consumes lots of manpower and resources, can be addressed thoroughly. Front-line workers can be freed from the onerous tasks of inspecting the whole nearshore area and only need to focus on several key water areas where HABs accumulate as monitored (Figure 3 and Figure 4). Moreover, after the outbreak of HABs in lakes, nearshore areas usually become the places where HABs gather and secondary disasters occur. Considering this background, timely grasping of HAB information in nearshore areas is quite valuable for the prevention and control of HABs because disposal equipment for HABs, such as salvage equipment, can be allocated timely to key nearshore areas and the hazards caused by HABs can be decreased to the utmost extent.

Additionally, multi-source data on HABs and water quality can be integrated and visualized in the DTLF, which is quite valuable for situation analysis and judgment of HABs in lakes, because comprehensive information about HABs, e.g., area, spatial distribution, etc., can be acquired. Multi-dimensional analysis of water quality in the lake body can also be achieved, i.e., layered analysis and section analysis. This function can help water environment managers to grasp comprehensive information about lake water quality, for both the water surface and underwater spaces. This can improve the accuracy of situation analysis and judgment. For example, if there are no HABs gathering on the water surface but the concentration of Chl-a is high in the underwater space, it can be inferred that the risk of HAB occurrence is high in this area, and emergency plans can be developed in advance to reduce the potential harm.

### 3.2. Potential Applications of DTLF

Although applied in Lake Chaohu currently, the constructed DTLF is transferable to other lakes and reservoirs. Despite being data-driven, the DTLF developed in this paper is generic and therefore should be readily transferable to other lakes and reservoirs when the following conditions can be satisfied:

(1) There are available data sources from satellite remote sensing. As described in Section 5.2, area and spatial distribution of HABs in the whole lake are monitored with satellite remote sensing. And it is thus necessary that there are available data sources from satellite remote sensing. Considering the revisit cycles of satellites and the uncontrollability of meteorological conditions, there should be various data sources from satellites so that valid data on HABs in lakes and reservoirs can be obtained even when some satellites cannot obtain valid images. It can be accordingly inferred that the higher the number of data sources from satellite remote sensing, the better the application effect of the developed DTLF.

(2) There is a certain number of monitoring equipment. Firstly, land-based video monitoring devices equipped with visible light cameras are essential for obtaining the present situation of HABs in nearshore areas of lakes and reservoirs. Considering the effect of DTLF construction, i.e., the monitoring of HABs in nearshore areas, the number of video devices should be determined by the shape and length of the lakes, the heights of the video device, the performance of the imaging equipment, etc. Based on practical experiences, it is suggested to deploy one land-based video device around the lake every 5 km. Moreover, a certain amount of in situ monitoring systems is necessary to frequently acquire key water quality parameters, such as Chl-a concentration, in core positions of lakes and reservoirs. Additionally, at least one water level monitoring station is necessary for dynamical monitoring of water levels and calculating the lake surface, as explained in Section 5.1. All in all, the amount of monitoring equipment should be determined based on the areas and shapes of the lakes and reservoirs. And the more monitoring equipment and the more uniform their spatial distribution, the better the application effect for the developed DTLF.

(3) There is detailed basic data materials for lakes (reservoirs) and watersheds. As described in Section 5.1, the construction of a DTLF needs support from basic data materials such as 3D terrain data for the watershed and a 3D model of the underwater topography of lakes.

### 3.3. Uncertainties in the DTLF and Future Work

Although the constructed DTLF has several striking advantages as discussed in Section 3.1, it is important to note that there are several existing weakness/uncertainties. Some of them will require improvements in the future.

(1) Video devices around lakes and reservoirs are presently exploited mainly for several purposes, such as illegal fishing monitoring, security monitoring, etc., and it is quite difficult to keep the characteristics of the devices stable, resulting in uncertainties in HAB monitoring results. For example, for a fixed water area where HABs accumulate, the obtained coverage ratios of the HABs can be different by adjusting the angle of the video device;

(2) In this DTLF, the present condition of HABs in nearshore areas can be obtained rapidly, automatically and periodically according to coverage ratios of HABs monitored with land-based video devices and the Kriging Interpolation method. Although this is quite valuable for HAB management practices, there are several factors which can affect the obtained present condition of HABs, such as the uncertainty of HAB distribution, the limited monitoring ranges and the multi-purpose nature of the video devices, etc.

(3) Although the DTLF can be constructed and dynamically refreshed with dynamically monitored water levels as explained in Section 5.1, the used water levels were assumed to be planar, which is not reasonable, especially in large lakes or reservoirs. Moreover, the waves were not used when constructing the DTLF, and there is a certain disconnect between the DTLF and the real lake body.

## 4. Conclusions

A novel digital twin lake framework (DTLF) was constructed in this study for HAB monitoring and management. Our application case in Lake Chaohu revealed that the constructed DTLF can obtain HAB information in nearshore areas frequently (once per second from 8 a.m. to 6 p.m. every day). Moreover, multi-source monitoring data on HABs and water quality can be integrated, represented and analyzed in a 3D manner, and the comprehensive situation of HABs and water quality in lakes can be obtained. More basic data materials, monitoring data on the water level and effective methods for wave simulation may help to improve the effects of DTLF construction. In addition, fixed spatial characteristics of land-based video devices can significantly improve the certainty and practicality of the results of HAB monitoring. Accordingly, we will pay more attention in future work to collect more basic data materials, simulate waves and structure video monitoring systems around lakes and reservoirs with fixed imaging postures.

## 5. Materials and Methods

### 5.1. Construction of the DTLF

**Figure 8:**
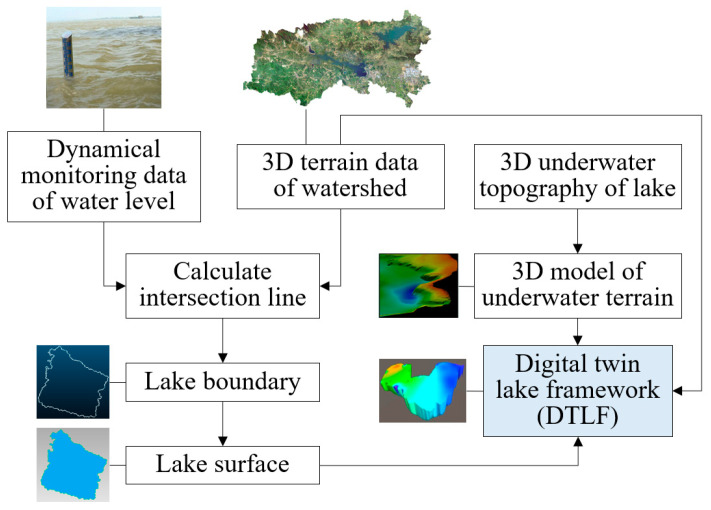
The process of DTLF construction. Note that the monitoring data on the water level are dynamic and thus the constructed DTLF is time-varying.

In this paper, a DTLF is defined as the digital counterpart of a real lake body. In theory, information of HABs and the water quality of a lake can be freely integrated, represented and analyzed in its DTLF. A DTLF of a lake can be constructed with 3D terrain data on the watershed, water level and 3D underwater topography of the lake. For a specific lake, the process of DTLF construction (Figure 8) can be summarized with the following three steps:

(1) Dynamically determine the lake boundary and the lake surface. It can be inferred that the lake boundary and the lake surface vary with the changes of water level. In this work, a planar model is firstly generated according to the dynamically monitored water levels of the lake; the lake boundary is then determined by calculating the intersection line of the determined planar model and the 3D terrain data for the watershed. And the enclosed space of the determined lake boundary is considered as the lake surface;
(2) Generate the 3D model of the underwater topography of the lake. A batch program is implemented and applied which can assign heights for all points in the underwater topography according to their elevations. And then the 3D model of the underwater terrain can be generated by fitting a curved surface based on all processed points;
(3) Generate the final DTLF. Merge the obtained lake surface and the 3D model of the underwater terrain of the lake to generate the final DTLF. Considering the dynamic character of the water level, an algorithm is designed to refine the generated DTLF, i.e., (i) if the boundary of the 3D model of the underwater terrain is lower than the lake surface, i.e., there are empty areas between them, mend the empty areas with the 3D terrain data for the watershed, and (ii) if the boundary of the 3D model of the underwater terrain is higher than the lake surface, clip the 3D model of the underwater terrain based on the lake surface, i.e., only keep 3D points with heights less than or equal to the height of the lake surface.

### 5.2. HAB Monitoring throughout the Whole Lake via Satellite Remote Sensing

The FAI index [29] was used in this paper for HAB monitoring based on satellite imageries including Terra/Aqua MODIS, Sentinel-2 MSI and GOCI. In addition, a satellite-based automatic method for HAB monitoring was designed and applied which can be seen in detail in [17]. With this method, the whole process of satellite-based HAB monitoring was completed without any artificial participation.

### 5.3. Video-Based Real-Time Monitoring of HABs in Nearshore Area of Lake

**Figure 9:**
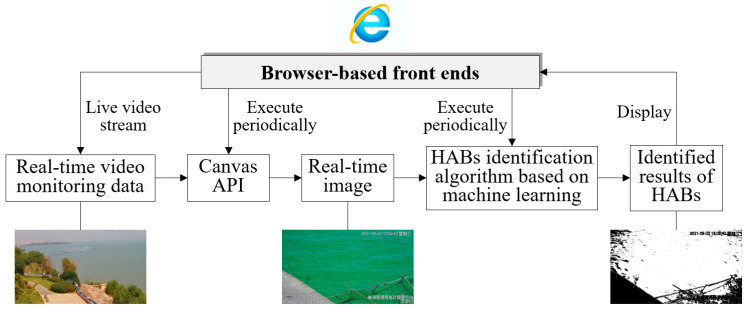
The basic schematic diagram of video-based real-time monitoring of HABs in nearshore areas of lakes. Note that only browser-based front ends are used to display live video data, capture real-time images, identify HAB pixels and display the results of HAB monitoring, and the efficiency of HAB monitoring can be improved significantly because data transfer processes between front-sides and server-sides are avoided.

After the outbreak of HABs in lakes, nearshore areas usually become the places where HABs gather and secondary disasters occur. Therefore, real-time monitoring of HABs in nearshore areas is quite valuable but the existing research cannot achieve this goal. In this paper, a video-based real-time monitoring method for HABs was designed (Figure 9) which can be summarized with the following three steps:

(1) Obtain real-time images from video monitoring data via Canvas API [39], a new tag added in HTML5 for generating real-time images on web pages and manipulating image content. Canvas API is executed periodically with browser-based front ends and the period can be set as needed;

(2) Develop a machine-learning-based algorithm for HAB identification. U-Net [40], a semantic segmentation model of digital images which can be trained with a small dataset, is used in this paper for HAB identification. Before formal application, the U-Net model was designed and trained via the following six sub-steps, i.e., (i) prepare original images captured with land-based video devices and the corresponding handmade label images (classify image pixels into HAB pixels and HAB-free pixels), and then divide these images into two groups, i.e., training data and validation data, (ii) preprocess the original images and label the images, such as cropping, scaling, normalization, etc., (iii) design the U-Net model, including the encoder and decoder, (iv) use the training data to train the designed U-Net model, including forward propagation, backpropagation and parameter updates, (v) use the validation data to validate the model and evaluate its performance and effectiveness and (vi) optimize the model based on the validation results, such as adjusting hyperparameters, adding training data, etc.;

(3) Dynamically calculate the results of HAB monitoring. After each instance of HAB identification, the coverage ratio of HABs, i.e., the ratio of HAB pixels to the total pixels, is calculated. And the present condition of HABs in the whole nearshore area can then be obtained with spatial interpolation methods such as Kriging Interpolation.

### 5.4. Integration, Visualization and Analysis of Multi-Source Monitoring Data

(1) Integration and visualization of multi-source monitoring data. Based on the constructed DTLF, display the HAB data monitored with satellites, video devices and in situ stations in a multi-layer-overlay manner. To prevent multi-source HAB information from being obstructed, results of in situ monitoring, satellite remote sensing and video monitoring are placed in the back layer, the middle layer and the top layer, respectively;

(2) Multi-dimensional analysis of water quality. Based on the constructed DTLF, the spatial distribution of water quality can be expressed in a 3D manner via spatial interpolation. And multi-dimensional analysis of water quality can be realized in the whole lake body, such as layer representation (visualize the horizontal distribution of water quality in different depths), section representation (visualize the vertical distribution of water quality in different sections), etc.

### 5.5. Performance Validation

(1) Accuracy validation. Firstly, considering that the outbreak of HABs in lakes is closely related to the concentration of Chl-a in water bodies, Chl-a concentration obtained via in situ monitoring is used in this paper to validate the accuracy of satellite remote sensing. Moreover, F-score [41] is used to quantitatively assess the accuracy of video monitoring, which is defined as follows:

(1) $$\left\{\begin{array}{l} F=\frac{2\times P\times R}{P+R}\\ P=\frac{TP}{TP+FP}\\ R=\frac{TP}{TP+FN} \end{array}\right.$$

where TP (true positive) is the number of correctly identified HAB pixels, FP (false positive) is the number of HAB-free pixels which are identified as HABs, FN (false negative) is the number of HAB pixels which are identified as HAB-free, P (precision) is the proportion of correctly identified HAB pixels against identified HAB pixels, R (recall) is the proportion of correctly identified HAB pixels against true HAB pixels and F (F-score) is a balance of P and R, which is one of the most useful metrics to evaluate the performance of HAB extraction with land-based video monitoring devices;

(2) Efficiency validation. Considering the dynamic variations of HABs, efficiency is a significant evaluation index for HAB monitoring methods. In this paper, consumed time is taken as the index for efficiency evaluation;

(3) Effect validation. In this paper, effect validation contains two aspects, i.e., analysis function and visualization effect. The former verifies whether the designed functions, such as multi-dimensional water quality analysis, are implemented, and the latter validates the effect of multi-variate and multi-dimensional data integration and representation.

## Figures and Tables

**Table 1:** The server configuration for efficiency evaluation.

CPU	RAM	Bandwidth	OS	Hard Disk Space
Intel Core i7-11370H	32 GB	8 Mbps	Windows Server 2019	2048 GB

**Table 2:** The results of efficiency evaluation.

ID	Test Case	Consumed Time	Remarks
1	Land-based video monitoring	0.1 s	Contains the processes of real-time image capturing, HAB pixels identification and HAB monitoring results expression
2	Satellite remote sensing	176 min	Contains the processes of satellite imagery download, data processing, result storing and data distribution. And the actual efficiency may be different in different network environments.

## Data Availability

The data and materials that support the findings of this study are freely available upon request from the corresponding author (corresponding author’s e-mail: ygqiu@niglas.ac.cn).
